# The Appropriateness and End Outcomes of Urgent Referrals to Outpatient General Adult Psychiatry

**DOI:** 10.1192/bjo.2024.386

**Published:** 2024-08-01

**Authors:** Zoe Johnston, Douglas Murdie, Kenneth Murphy

**Affiliations:** NHS Lothian, Edinburgh, United Kingdom

## Abstract

**Aims:**

The urgent referral system to outpatient psychiatry in NHS Lothian is intended for patients who require to be seen within 5 days. However, many of the referrals are not deemed this urgent upon triage. This project aims to illustrate the extent of this issue and highlight potential reasons, in order to improve the pathway for patients referred on to secondary care services.

**Methods:**

Over a 3 month period from August 2023 to November 2023, all urgent referrals received by an Edinburgh sector general adult psychiatry outpatient's department were reviewed. Data was collected on broad presenting complaint, whether or not the referral was deemed urgent upon triage, whether it contained a factor in line with RefHelp guidance for urgency, and what the end outcome of the referral was.

**Results:**

During the 3 month period, there were 92 urgent referrals. Of these, only 12% were deemed urgent upon triage. Almost all accepted referrals related to concerns around potential psychotic illness (82%). Although only 12% of referrals were accepted as urgent, 35% had factors which, in accordance with RefHelp guidance, would be cause for considering an urgent referral.

There were a variety of disposals including “soon” appointments, redirection to other services such as Thrive or offering advice to the referring clinician. The most common outcome was the offer of a “soon” appointment, closely followed by redirection to the Thrive team.

**Conclusion:**

The majority of urgent referrals were not deemed urgent at triage. There was a clear discrepancy between referrals containing urgency factors according to RefHelp and those offered urgent appointments. This would suggest that the available guidance is not sufficiently clear.

Many referrals were redirected to other services, including Thrive. This redirection may reflect a lack of awareness and a further project may examine Thrive referrals to establish if the number initially sent to psychiatry outpatients is significant.

Additionally, several referrals were triaged as “soon” and seen in 6–8 weeks, as opposed to waiting for a routine appointment. Though RefHelp advises highlighting routine referrals which may be a priority, this pathway was not being used and there is no direct route for “soon” referrals.

Next steps may include liaison with primary care teams to establish views and concerns, updating RefHelp guidance and adding a further referral pathway to address the apparent gap for “soon” referrals.

